# The association between education and induced abortion for three cohorts of adults in Finland

**DOI:** 10.1080/00324728.2015.1083608

**Published:** 2015-10-08

**Authors:** Heini Väisänen

**Affiliations:** ^a^ London School of Economics and Political Science

**Keywords:** induced abortion, register data, Finland, reproductive health, event-history analysis

## Abstract

This paper explores whether the likelihood of abortion by education changed over time in Finland, where comprehensive family planning services and sexuality education have been available since the early 1970s. This subject has not previously been studied longitudinally with comprehensive and reliable data. A unique longitudinal set of register data of more than 250,000 women aged 20–49 born in 1955–59, 1965–69, and 1975–79 was analysed, using descriptive statistics, concentration curves, and discrete-time event-history models. Women with basic education had a higher likelihood of abortion than others and the association grew stronger for later cohorts. Selection into education may explain this phenomenon: although it was fairly common to have only basic education in the 1955–59 cohort, it became increasingly unusual over time. Thus, even though family planning services were easily available, socio-economic differences in the likelihood of abortion remained.

## Introduction

In many countries women in less advantaged socio-economic positions have more abortions than other women (Jones et al. 2002; Rasch et al. 2007; Hansen et al. 2009; Regushevskaya et al. 2009). High prevalence of contraceptive use has been shown to reduce the number of abortions in a population (Bongaarts and Westoff 2000) and healthcare costs (Frost et al. 2008, 2014; Cleland et al. 2011), but studies have not examined whether universal access to family planning services reduces socio-economic differences in the likelihood of abortion.

The aim of the study reported in this paper was to investigate differences by education in the likelihood of abortion in Finland, a country where comprehensive family planning services and sexuality education have been available since the early 1970s (Kosunen 2000; Kontula 2010), and where parents are offered generous financial and other help to enable them to ensure that at least the essential needs of their children are met (Vikat 2004; Haataja 2006). The study used a unique and nationally representative longitudinal data set based on administrative registers that made it possible to investigate both the association between education and the likelihood of abortion and—something that to the best of my knowledge other studies in Finland or elsewhere have been unable to investigate—whether the association changed over time. The nature of the data set meant that the study did not suffer from attrition and non-response common in panel studies or the common problem of underreporting of abortions in surveys (Jones and Kost 2007).

Previous studies on the topic  in Finland differed from the one reported here in one or more of the following respects: they were based on cross-sectional surveys (Regushevskaya et al. 2009); they studied women who had had at least one abortion, thus ignoring those who had never experienced one (Heikinheimo et al. 2008, 2009; Niinimäki et al. 2009; Väisänen and Jokela 2010); they did not investigate the women's level of education (Vikat et al. 2002; Hemminki et al. 2008; Sydsjö et al. 2009). Because most other countries in which studies have been undertaken do not have register data on abortions (Gissler 2010), their studies have been based on surveys, which often suffer from underreporting of abortions (Jones and Kost 2007).

### Socio-economic status and pathways to abortion

Previous studies in the US and Europe (including Finland) have shown that the likelihood of having an abortion is positively associated with the following characteristics: low socio-economic status (SES) (Rasch et al. 2007; Väisänen and Jokela 2010; Klemetti et al. 2012); low education and income (Jones et al. 2002; Regushevskaya et al. 2009); young age (Jones et al. 2002; Knudsen et al. 2003; Rasch et al. 2007; Niinimäki et al. 2009; Klemetti et al. 2012); being single, having relationship problems, or previous births (Jones et al. 2002; Rasch et al. 2007; Hansen et al. 2009; Regushevskaya et al. 2009; Klemetti et al. 2012); and previous abortions (Hansen et al. 2009; Niinimäki et al. 2009).

A higher likelihood of experiencing an unintended pregnancy is associated with a higher likelihood of an abortion. Unintended pregnancies may be unwanted (not wanted at all) or mistimed (preferred later) (Trussell et al. 1999; Santelli et al. 2009). Pregnancies may be unintended for one or more of the following reasons: because a woman does not want to have any (more) children, because she wants to postpone childbearing, because she does not want to have children with her current partner, or because she perceives her socio-economic situation as unfavourable for childbearing.

Low education and income have been associated with a higher likelihood of unintended pregnancies in the US (Finer and Zolna 2011), the UK (Wellings et al. 2013), and Spain (Font-Ribera et al. 2007). That association was not found in a study in the Netherlands. Although highly educated women there were overall found less likely to become pregnant, there was no association between education and the proportion of unintended pregnancies among all pregnancies (Levels et al. 2010).

Contraceptive failure or lack of contraceptive use when there is no intention to become pregnant, may lead to an unintended pregnancy. Studies have found that higher SES is associated with more effective contraceptive use and more satisfaction with family planning services in the US (Ranjit et al. 2001; Frost et al. 2007; Kost et al. 2008) and Finland (Hemminki et al. 1997; Kosunen et al. 2004).

### The Finnish context

Before 1970, legislation in Finland allowed abortion in the following circumstances only: the woman's life or health was at risk, or one of the parents was believed to have a severe physical or mental illness, or the foetus had a medical problem, or pregnancy was due to rape or incest, or the woman was younger than 16 (Keski-Petäjä 2012). A change of legislation in June 1970 established more liberal provisions. In particular, the change allowed abortion for ‘social reasons’, defined as being under considerable strain owing to any of the following: living conditions or other circumstances; being younger than 17 or older than 40; and already having at least four children (Knudsen et al. 2003). At first, abortions for most social reasons were allowed until the end of 16 weeks’ gestation, but in 1978 that was changed to 12 weeks. If the woman is younger than 17, or there is another special social reason for abortion, an abortion can be allowed until the end of 20 weeks’ gestation. It is allowed until the end of 24 weeks’ gestation if the foetus has a medical problem, and there is no limit on the period of gestation if the woman's life or health is at risk. If the abortion is sought because of ‘considerable strain caused by living conditions or other circumstances’, the approval of two doctors is required. If it is sought on the grounds of a woman's age or number of children, the approval of one doctor is enough (Knudsen et al. 2003). In practice, approval is granted if a woman applies for an abortion before the end of 12 weeks’ gestation (Gissler 2010).

Attitudes towards abortion are liberal in Finland: 65 per cent of Finns believe that abortions should be available on request (Kontula 2008). In the early 1990s, only 5 per cent of Finnish women were against abortion in all situations (Notkola 1993).

Abortions are currently provided at low cost in the public healthcare sector—for example, one of the hospital districts charges between €30 and €100 depending on the duration of the pregnancy and whether it is a medical or surgical termination (YTHS 2014). Financial help is available for those unable to pay.

Although all municipalities have been required by law to provide family planning services since the 1972 Primary Health Care Act, access is not necessarily equal for women in all SES groups. First of all, women have to pay for contraceptives. Condoms have low one-off costs, oral contraceptives cost €60–150 per year, and intra-uterine devices (IUDs) about €80–150 when inserted (Koistinen 2008; Väestöliitto—Family Federation of Finland 2012; University Pharmacy 2014). These figures are roughly equal to about half of 1 per cent of women's median annual income in the private sector in 2010 (Statistics Finland 2011). Although the cost is low, it may still pose an obstacle for someone at the lower end of the income scale. Another obstacle for some is lack of timely access to family planning services. Public clinics provide free or affordable services, but have long waiting times. Private clinics have shorter waiting times and more often offer appointments with specialists, but are expensive. The private clinics are more often used by high-SES than low-SES women (Hemminki et al. 1997).

There have been few studies of contraceptive use by education in Finland, but a nationwide survey of women aged 18 to 44 in 2000 found that women with university-level education were twice as likely to use oral contraceptives as women with basic education (21 per cent vs. 12 per cent), but that almost 20 per cent of women in both groups used IUDs (Kosunen et al. 2004). Unfortunately, these figures were not adjusted for age or any other covariate and condom use was not reported. The study also found that 36–48 per cent of those aged 18 to 44 used oral contraceptives, whereas around 25 per cent of women aged 35–44 relied on IUDs and only 2–13 per cent on oral contraceptives (Kosunen et al. 2004).

Women have relatively few abortions in Finland. The total abortion rate (TAR), which is the expected number of abortions a woman would have if the age-specific abortion rates observed in a given year continued throughout her entire fertile period of life, decreased from 0.4 in 1980 to 0.3 in the mid-1990s, where it has since remained. (I calculated the rate from the number of abortions in 5-year age groups (Vuori and Gissler 2013) and the number of women in each age group (Official Statistics of Finland 2013b).) It is one of the lowest TARs in Europe and North America. For instance, in the 1990s and 2000s the TAR for England and Wales was around 0.5, for the US around 0.6, and for Russia higher than 1 (Sedgh et al. 2013). Lower TARs than in Finland have been observed, for example, in the Netherlands, Belgium, and Germany (all between 0.19 and 0.27 in the period 1995–2009) (Sedgh et al. 2013).

The total number of abortions in Finland decreased from 21,547 in 1975 to 9,872 in 1995. Since 2000, there have been around 11,000 abortions per year (Vuori and Gissler 2013). The abortion rate per 1,000 women of fertile age, which was 18 in the mid-1970s, decreased steadily to around 9 for the period from 2000 to the present (Gissler and Heino 2011; Vuori and Gissler 2013).

### The aim of the study

In the study reported here, I focused on the likelihood of first abortion by education level for women who chose to terminate a pregnancy on social grounds, which are the grounds cited for more than 90 per cent of all abortions in Finland (Vuori and Gissler 2013). The specific research questions were as follows. How strong is the association between education and the likelihood of abortion? Has the strength of the association changed over time? Has the increasing level of education in the population been associated with changes in abortion rates? The results of previous studies led me to expect low education to increase the likelihood of abortion (Jones et al. 2002; Regushevskaya et al. 2009), but offered no guidance on whether better information on contraceptive use and access to family planning services and sexuality education were likely to be associated with differences by education in the likelihood of abortion. It seemed possible that as more women had better information on contraceptive use and access to family planning services, differences by education would decrease. On the other hand, if it was the more educated women who had taken advantage of easier access to these services, the effect could have been to increase the differences by education in the likelihood of abortion. Other studies have shown that it is typically people of higher SES who are the first to take advantage of new public services, and thus benefit disproportionally from them (Hemminki et al. 1997; Watt 2002; Saurina et al. 2012).

The majority of abortions in Finland are first abortions (63–73 per cent of all abortions in the period 1987–2010 (Vuori and Gissler 2013)), and this was the category chosen for the study. The determinants of these may differ from those that explain higher-order abortions. For instance, it has been reported that women seeking their second or higher-order abortion have lower education than those seeking first abortions (Jones et al. 2006; Makenzius et al. 2011) and are more likely to use barrier methods and oral contraceptives than long-acting reversible methods (Osler et al. 1997; Jones et al. 2006; Heikinheimo et al. 2008; Niinimäki et al. 2009). The study was restricted to women aged 20 to ensure that all the subjects of the study were old enough to have completed at least basic education. Many had completed upper secondary, which is typically completed by age 20 in Finland, but enough had not done so to allow a comparison between these groups. More clear-cut findings were possible for women aged 25 or over because many in their early 20s had not yet finished their education, while women aged 25 or more were likely to have achieved the highest level of education they would attain. Moreover, the circumstances in which adult women choose to have an abortion often differ from those in which teenagers do so. The costs of childbearing for the latter are more severe because they may not have completed their education or formed stable partnerships or had time to accumulate resources (Becker 1991; Oppenheimer 1994; Hansen et al. 2009; Kreyenfeld 2010; Väisänen and Murphy 2014). Another reason for not studying the association between family SES and the likelihood of abortion among Finnish teenagers was that this had already been the subject of a study by Väisänen and Murphy (2014).

## Data

Nationally representative data on three birth cohorts of females (1955–59, 1965–69, and 1975–79) were obtained in anonymized form from the Registry of Induced Abortions, the Medical Birth Registry, and the Population Registry of Finland (for a comprehensive description of these registries, see Gissler et al. 2004, p. 423). Statistics Finland linked these registries using a unique identification number held in Finland for each permanent resident. Evaluation studies have found registers to be reliable sources of information (Gissler et al. 1996; Gissler and Shelley 2002).

The data were selected using two-stage sampling. First, an 80 per cent random sample of all the women in the above-mentioned cohorts who had had at least one abortion within their fertile period (assumed to be ages 15–50) was selected (*N* = 91,636). Because some of the women had not reached age 50, they were included in the sampling frame if they had had an abortion before the end of 2010, the end of the study period. The reason why all women from these cohorts who had ever had an abortion were not included in the data is that Statistics Finland do not allow the use of complete (sub-)populations for research purposes, on ethical grounds. Second, a comparison group, twice the size of the abortion group, of women from the same cohorts who had not had an abortion were selected using random sampling (*N* = 183,272). The sample was taken from women who had lived in Finland for at least a year (although most of these women had spent all their lives in Finland) within any of the following periods: 1970–75, 1980–85, or 1987–2010 and had not had an abortion during their time in the country. These periods were chosen because they were the years when detailed census information on the Finnish population was available. In the statistical analysis, weights were used to control for this design. Overall, the unweighted sample included almost half of the women of these three cohorts.

Because this was a study of adult women, those in the original sample who had died (*N* = 621) or emigrated (*N* = 5,233) before age 20 were not included. It was assumed that someone had emigrated if there was some information in the registers about her, but none after a certain point and no year of death was recorded. Most women entered the study when they reached age 20, but the 13,308 women who immigrated when aged 21 or older were included in the sample on their year of arrival in Finland. Overall, 269,054 women were included in the study. The number changed over time owing to mortality and migration. There were 91,636 first abortions in the data, 65,384 of which took place at age 20 or later. Of these abortions, 62 were recorded as having taken place before the woman's recorded year of immigration and were therefore excluded from the analyses. Of the remaining abortions, 58,183 were conducted for social reasons, 6,018 for medical reasons, and 1,121 for reasons that were not recorded.

The data set included information on the following: induced abortions; live births; education (basic, upper secondary, further, undergraduate, postgraduate); occupational group (manual worker, upper-level or lower-level non-manual employee, farmer, self-employed, student, other); place of residence (urban, semi-urban, rural, and province—South, West, East, North, Lapland, and Western Archipelago); immigration status (whether born in Finland and whether native language is one of the official languages, i.e., Finnish or Swedish); and relationship status (single, cohabiting, married (including separated women because they are grouped together in the population register), divorced, widowed).

Statistics Finland does not give detailed information for research purposes about people with less than upper secondary education and codes their education status as ‘missing’. In such cases I assumed that the woman had received basic education only. Basic education lasts on average 9 years, and upper secondary typically a further 3 years. ‘Further education’ means schooling after upper secondary education that has not led to an undergraduate or postgraduate degree.

Year and month of abortions and live births are shown in my data set. Changes in marital status are updated once a year. Cohabitation was not recorded before 1987 but has since been recorded annually. In my data set, place of residence, occupational group, and level of education were recorded at ages 20, 25, and 30 or the nearest year possible, because information on education and place of residence was recorded in the Population Register every 5 years (census years 1970, 1975, etc.) until year 1987, and until 2004 for occupational group, and then annually. For the statistical analysis, I used the latest information of socio-economic data available; for instance, the value recorded at age 20 was used until new information recorded at age 25 was available.

## Methods and analytical strategy

The analysis proceeded as follows. I calculated the number of first abortions by reason (social or medical) per 1,000 women by age, education, and cohort to see whether the numbers differed by these characteristics. The denominators included women who had already had an abortion, although they were no longer at risk of having their first abortion, since these rates have conventionally been based on the whole population.

In order to assess whether the differences in abortion by education changed over time, I calculated concentration curves of education and the incidence of abortion using aggregate data. I plotted weighted cumulative percentage of abortion against cumulative level of education beginning from the lowest level (see, e.g., Chen and Roy 2009; Konings et al. 2009; Erreygers and Van Ourti 2011). With this method, if abortions are equally distributed among education groups, the concentration curve coincides with the 45° ‘equality line’. The further the concentration curve is above the equality line, the more common are abortions among the less than the more educated women (Chen and Roy 2009; Erreygers and Van Ourti 2011). Since level of education was an ordinal variable with five categories unequally distributed within the population, I had to assume that the distribution of abortion was constant within education groups (Konings et al. 2009), although it may not have been. Since the data included 80 per cent of women who ever had an abortion, the estimates were precise and it was unnecessary to provide confidence intervals.

In order to explore whether changes in abortion rates across cohorts were attributable to the changing educational composition of the population, I calculated standardized cohort abortion rates by age group (20–24, 25–29, 30–34) and cohort, using the distribution by education of the 1950s cohort as standard. This shows the expected number of abortions per 1,000 women for the other two cohorts had their distribution by education been the same as that of the 1950s cohort (see, e.g., Hinde 1998). Comparing the standardized estimates with those observed reveals whether abortion levels would have been different had the educational composition of the population not changed, all else being equal.

Discrete-time event-history analyses were used to determine whether the patterns by education held after controlling for other factors known to be associated with the likelihood of abortion. The following control variables were included: parity, months since last birth and its quadratic term, indicator of being childless (0 for women with no live births recorded, 1 for others), place of residence, occupational status, relationship status, and immigration status (Jones et al. 2002; Vikat et al. 2002; Rasch et al. 2007; Hansen et al. 2009; Regushevskaya et al. 2009).

Discrete-time event-history models are logistic regression models with time included as a dummy variable; in this case time was measured as age because year-wide increments centred around age 20, the start of the study period. The women were followed until their first abortion for social reasons or censored at whichever of the following occurred first: end of year 2010, age at emigration, death, age 50, or an abortion for either a medical reason or without a recorded reason. In order to allow for differences in the estimates by age and cohort (Steele et al. 2004), the analyses were run separately for the three cohorts and 5-year age groups (20–24, 25–29, 30–34, 35+). Another analysis used as a robustness check, estimated a model that included all cohorts in which education was interacted with cohorts and age groups to test the statistical significance of the interactions. I chose discrete-time models because including time-varying covariates in them is straightforward (Steele et al. 2004) and the implicit assumption that the hazard function and covariate values are constant within each 1-year age interval leads to a minimal loss of information compared with continuous time models such as Cox regression (Steele et al. 2005).

To show the results of the event-history analyses, I calculated fitted probabilities of abortion by age group and level of education, using average marginal effects at representative values. This entailed treating all respondents as though they had the level of education of interest, say basic education, leaving the values of all other variable as observed when calculating the probability of abortion. The same calculation was conducted for each of the five levels of education. The average of these marginal effects became the probability of having an abortion in each education and age group (Williams 2012). I present the results as the predicted number of abortions per 1,000 women, with 95 per cent confidence intervals.

All of these estimates highlighted a slightly different aspect of the association between education and abortion. The fact that they all pointed to the same interpretation of the association between education level and abortion was a good indication of the robustness of the results. Stata 13 was used for all analyses except the concentration curves, which were calculated using R 2.15.

## Results

As Table 1 shows, half the women in the 1950s cohort had only basic education at age 20, but the proportions had fallen to only around a quarter in the 1960s and 1970s cohorts. By age 30, a quarter of women still remained in this category in the earliest cohort, but only 11–15 per cent in the other two cohorts. Also, the proportion of women with an undergraduate or postgraduate degree by age 30 was higher for the 1970s cohort (42 per cent) than in the other cohorts (10 and 15 per cent in the 1950s and 1960s cohorts, respectively).

**Table 1:** Women's level of education at ages 20, 25, and 30 by cohort in Finland, weighted percentage and unweighted *N*

		1955–59			1965–69			1975–79		
Variable	Category	20^1^	25^1^	30^1^	20^1^	25	30	20	25	30
Education	Basic	47.9	27.7	24.1	23.2	16.8	15.2	18.2	12.5	11.2
	Upper secondary	47.3	47.7	39.1	75.2	69.7	48.8	54.1	53.7	38.7
	Further	4.8	17.1	26.5	1.6	7.5	20.7	27.7	10.4	8.5
	Undergraduate	0.0	5.4	4.6	0.0	2.6	4.3	0.0	17.6	24.8
	Postgraduate	0.0	2.1	5.7	0.0	3.3	11.0	0.0	5.9	16.8
	Total = 100% (*N*)	(102,014)	(101,090)	(100,442)	(95,540)	(96,102)	(96,439)	(58,173)	(58,746)	(59,149)

^1^Measured at age 20, 25, or 30 or the nearest year possible (see text).

*Source*: Register data from Statistics Finland and the National Institute for Health and Welfare.

**Figure 1:**
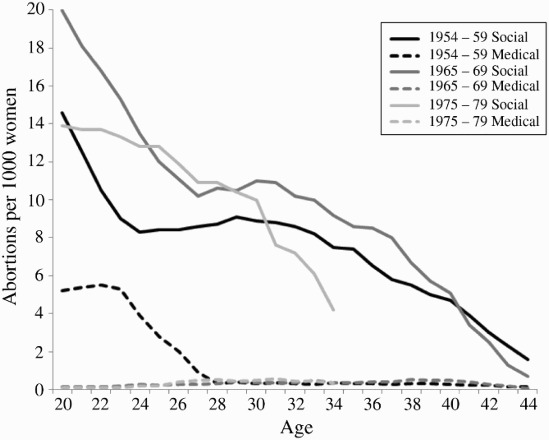
First abortion rates per 1,000 women by age, cohort, and indication of abortion (social or medical) in Finland

*Source*: Register data from Statistics Finland and the National Institute for Health and Welfare.

There were relatively more abortions—2–5 per 1,000 women—for medical reasons in the 1950s cohort among women younger than 27 years than in the other two cohorts (less than 1 per 1,000). This might be because legislation permitting abortion for social reasons came into force in June 1970 and it took time for the practice of recording this as the reason to become established (Figure 1).

**Figure 2:**
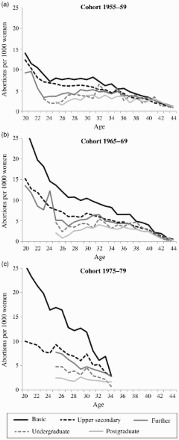
The number of first abortions for social reasons per 1,000 women of the same age and education group in Finland

*Source*: As for Figure 1.

Figure 2 shows that the first abortion rate varies across levels of education in all cohorts. Overall, differentials were largest for young women but decreased with age. Women with basic education had the highest abortion rate in all cohorts, but the differences were more pronounced in later cohorts. For instance, 20-year-olds in the 1950s cohort had 14 first abortions per 1,000 women if they had basic education, but 12 if it was upper secondary. In the 1960s cohort the corresponding figures were 28 and 15, and in the 1970s cohort, 26 and 10. Women with at least an undergraduate degree had low abortion rates—not more than 7 per 1,000—across all age groups and cohorts. The estimates for young women in the 1950s cohort may be biased downwards owing to the high number of abortions recorded as being for medical reasons. As stated earlier, this number may have been inflated by a delay in establishing the practice of recording social reasons as the actual reasons given (see Figure 1).

**Figure 3:**
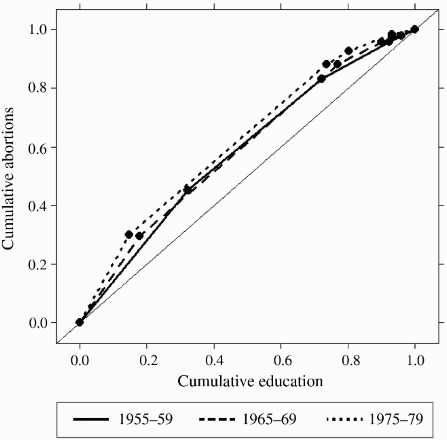
Concentration curves of the incidence of first abortion for social reasons against cumulative level of education by cohort in Finland

*Source*: As for Figure 1.

Figure 3 confirms that even when the changing educational composition of the population is taken into account, differences by education level in the likelihood of abortion increased for later cohorts. The 1970s cohort's curve is furthest away from the ‘equality line’, indicating that differences by level of education in the likelihood of abortion for that cohort was higher than for the other two. For instance, 20 per cent of women at the lower end of the education distribution had about 28 per cent of abortions in the 1950s cohort, 31 per cent in the 1960s cohort, and 35 per cent in the 1970s cohort.

The cohort abortion rate standardized for education level shows that part of the decline in the number of abortions was attributable to the changing distribution by education in the population. Had the distribution been the same for the 1960s and 1970s cohorts as it was for the 1950s cohort, more abortions would have occurred, all else being equal. The proportions per 1,000 women for the 1950s cohort were 9.6 for 20- to 24-year-olds, 6.2 for 25- to 29-year-olds, and 5.5 for 30- to 34-year-olds. Had the education distribution been the same for the 1960s cohort as it was for the 1950s cohort, there would have been 16.9 (instead of the observed 13.8) abortions per 1,000 women in the 20–24 age group, 7.9 for 25- to 29-year-olds (observed 7.1), and 6.4 for 30- to 34-year-olds (observed 6.0). For the 1970s cohort the standardized figure per 1,000 women in the 20–24 age group was 15.8 (observed 11.2), 9.5 for 25- to 29-year-olds (observed 7.4), and 5.2 for 30- to 34-year-olds (observed 4.3).

The adjusted event-history models also show that the higher the level of education, the lower the likelihood of abortion (Table 2). The association was stronger for the later cohorts than for the earlier ones and for younger women than for women in their 30s. For instance, women aged 20–24 with upper secondary education had 17, 39, and 51 per cent lower odds of abortion than women with basic education in the 1950s, 1960s, and 1970s cohorts, respectively, but the differences decreased by age. Women with university degrees had the lowest odds of abortion in almost all age groups and cohorts.

**Table 2:** Discrete-time event-history models for first abortion by age group and cohort in Finland. Hazard-odds ratios (HOR) with 95 per cent confidence intervals

Age	20–24		25–29		30–34		35+	
	HOR	CI 95%	HOR	CI 95%	HOR	CI 95%	HOR	CI 95%
Cohort 1955–59								
*Education*								
Basic (ref.)	1.00		1.00		1.00		1.00	
Upper secondary	0.83	(0.79–0.88)	0.83	(0.77–0.89)	0.79	(0.73–0.86)	0.95	(0.88–1.03)
Further	0.56	(0.48–0.66)	0.62	(0.55–0.68)	0.75	(0.68–0.83)	0.94	(0.85–1.03)
Undergraduate	0.34	(0.18–0.64)	0.47	(0.38–0.57)	0.71	(0.58–0.87)	0.90	(0.76–1.07)
Postgraduate			0.38	(0.27–0.54)	0.58	(0.47–0.71)	0.81	(0.68–0.96)
Cohort 1965–69								
*Education*								
Basic (ref.)	1.00		1.00		1.00		1.00	
Upper secondary	0.61	(0.58–0.64)	0.76	(0.70–0.82)	0.72	(0.66–0.80)	0.90	(0.81–1.00)
Further	0.55	(0.45–0.67)	0.61	(0.53–0.71)	0.68	(0.60–0.77)	0.75	(0.66–0.85)
Undergraduate			0.49	(0.38–0.64)	0.46	(0.36–0.58)	0.75	(0.62–0.91)
Postgraduate			0.27	(0.20–0.36)	0.48	(0.40–0.57)	0.58	(0.49–0.68)
Cohort 1975–79								
*Education*								
Basic (ref.)	1.00		1.00		1.00			
Upper secondary	0.49	(0.46–0.53)	0.64	(0.58–0.70)	0.84	(0.73–0.97)		
Further	0.40	(0.37–0.44)	0.57	(0.50–0.66)	0.71	(0.58–0.87)		
Undergraduate			0.40	(0.35–0.45)	0.55	(0.46–0.66)		
Postgraduate			0.26	(0.20–0.33)	0.41	(0.32–0.51)		

*Notes*: All models were estimated separately by cohort and age group, and include age, education, occupational group, indicator for being childless, months since last birth and its quadratic term, parity, relationship status, place of residence, and immigration status.

The model that included all cohorts and in which education was interacted with cohorts and age groups shows that the differences in the associations across cohorts and age groups were statistically significant at the 1 per cent level (results available on request).

**Figure 4:**
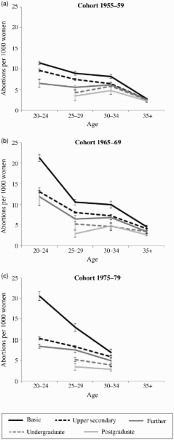
The number of abortions per 1,000 women by level of education and age in Finland with 95 per cent confidence intervals estimated using marginal effects at representative values. Adjusted for occupational group, indicator for being childless, months since last birth and its quadratic term, parity, relationship status, place of residence, and immigration status

*Source*: As for Figure 1.

Figure 4 shows the average marginal effects of differences in the calculated probability of abortion based on the event-history models (see Table 2). It shows the estimated number of abortions per 1,000 women by age and education group. Women with basic education have the highest probability of abortion in all age groups and cohorts, and the gap by level of education is wider for later cohorts than for earlier ones, especially among young women. For instance, there were on average 11 abortions per 1,000 women in the 20–24 age group in the 1950s cohort, and 21 in the 1960s and 1970s cohorts, but upper secondary education was associated with an average of 10–13 abortions per 1,000 women in this age group in all cohorts, and women with a university degree had fewer than 6 abortions per 1,000 women in all cohorts and age groups.

## Discussion

### Interpretation of the main findings

The results of this study show that providing ready access to family planning services and comprehensive sexuality education in schools does not eliminate differences by level of education in the likelihood of a first abortion. Women with only basic education had a substantially higher likelihood of first abortion than other women and the association was stronger for later cohorts. One explanation for this pattern is selection into education. Although it was still fairly common for women to have completed only basic education in the 1955–59 cohort, it became increasingly unusual in the later cohorts. Thus, women who have only basic education are probably different from other women in many other characteristics too. This explanation is supported by the fact that changes in the likelihood of abortion by occupational group were less dramatic than changes in its likelihood by level of education across cohorts (see Appendix). The occupational composition of the population changed less over time than the composition by education.

The cohort abortion rates standardized for education showed that it is likely that without the increase in education in Finland, relatively more abortions would have occurred in the later cohorts. Thus, part of the decline in abortion rates in the country is attributable to the changing educational composition of the population.

The differences by education level in the likelihood of abortion may arise partly because women with high education have better access to family planning services. Because waiting times are shorter in private clinics than in those provided by the public health service, and the former are more often used by high-SES women (Hemminki et al. 1997), it is possible that these women get more timely access to contraceptives than low-SES women. High-SES women may also have taken advantage more quickly than low-SES women of the new family planning services introduced since 1970 (Saurina et al. 2012). Another possible reason for the difference is suggested by a US study, which found that poorer women felt they had less choice over the contraceptive method they use, because some methods were too expensive (Cleland et al. 2011). Perhaps women with low education use less effective methods in Finland for similar reasons although differences are likely to be smaller than in the US because of the more generous financial support given to people with low income by the government. The study by Kosunen et al. (2004) showed that although use of IUDs was equally common across education groups, highly educated women more often than women with low education used oral contraceptives, indicating that contraceptive use does differ by education. In addition, highly educated women may use contraceptives more effectively because they have gained better knowledge of pregnancy prevention from their social networks (Kohler 1997). They may also be more literate in health matters, and thus better able to understand and critically assess (reproductive) health information (Nutbeam 2000).

If unintended pregnancies were equally common across all education groups, and the differences in abortion by education were due only to the differences in the likelihood of terminating a pregnancy, one would expect to see higher fertility levels among women with high education than among those with low education since highly educated women had fewer abortions. However, there are no large differences in completed family size by education in Finland (Andersson et al. 2009). It is thus more plausible that women with high education simply had fewer unintended pregnancies. This could be the outcome of differences in the frequency of sexual intercourse, but it is more likely that the differences in likelihood of abortion are explained by variation in contraceptive use.

### Strengths and limitations

This study was the first to analyse the association between education and the likelihood of abortion, using a large, representative and reliable longitudinal data set that was not suffering from drop-out or underreporting. Another useful feature of the data set was that it allowed the study to be restricted to those who had an abortion for social reasons (considerable strain caused by living or other conditions, being younger than 17 or older than 40, or already having at least four children) rather than for medical reasons (such as a medical problem of the foetus or a parent). This distinction is important because social and medical reasons may entail different decision-making processes: social reasons may be more often cited if the pregnancy was unwanted, whereas abortion may be necessary for medical reasons even if the pregnancy was wanted in the first place.

Although Finland is in many ways exceptional in the reproductive health services and family policies it provides for its population, the fact that the data set used for this study is richer and more reliable than those of most other countries (Jones and Kost 2007) may make the results of the study useful elsewhere. Reliable information on differences by level of education in the estimated likelihood of abortion is likely to be of interest to researchers and policymakers in other countries too.

The study had some limitations. The prevalence of abortions for medical reasons was higher among young women in the 1950s cohort than in the other cohorts, probably owing to delays in implementing change in the classification of reasons for abortion after the change in legislation in 1970. This may compromise the comparability of cohorts. However, when analyses were run using all abortions as outcome for the 1950s cohort, the interpretation of the model was essentially the same (results available on request).

The results obtained by concentration curves suggest that differences in the likelihood of abortion by level of education were higher for later cohorts, if one assumes that the distribution of abortion was constant within each education group (Konings et al. 2009). This assumption may be implausible. For instance, women who had completed years of university education, but had not (yet) graduated, were included in the upper secondary group together with women who never intended to pursue higher education. Moreover, although abortion rates standardized for education suggest that part of the decrease in abortion was attributable to a rise in the education level of the population, this inference is valid only on the assumption that all else was equal. Nevertheless, the results provide important descriptive information on how the association between abortion and education changed over time.

Another limitation of the study was that it lacked information on variables not included in registers and, owing to regulations intended to avoid providing information that could identify someone, important details on some variables that were included. Relevant information that was not available includes the woman's reason for choosing abortion, the partner's role in making the choice, pregnancy intentions, and contraceptive use. Also not available was information on factors known to affect the likelihood of an abortion, such as the attitudes and religious background of the women (Bankole et al. 1998).

Owing to the limitations of the data, it was not possible to investigate causal pathways to abortion. Nor was it possible to investigate whether obtaining education itself changes the women's likelihood of abortion or whether there are other unmeasured characteristics which make some women both more likely to obtain high education and less likely to have abortions. Women's contraceptive use, sexual activity, and willingness to terminate an unintended pregnancy affect their likelihood of having an abortion (Bongaarts 1978). These characteristics may depend on level of education, and thus partly explain the differences observed in this study. Since these characteristics were not measured in this study, their role could not be examined.

Despite the limitations, the strengths of the register data mean that the study was able to produce new and reliable information on the association between education and abortion over time.

## Conclusions

Analyses of register data on three birth cohorts of Finnish women (born in 1955–59, 1965–69, and 1975–79) over the reproductive period of their lives showed that differences by education in the likelihood of having an abortion increased over time. It would be useful if future studies used qualitative and survey data to investigate the effects of such variables as contraceptive use, pregnancy intention, and partner's characteristics in order to study the mechanisms causing the differences in the likelihood of abortion by education. It is important to ensure that all women, whatever their educational status, have easy access to affordable family planning services and know how to use contraceptives efficiently. Furthermore, use of long-lasting reversible contraceptive methods may help some women avoid unwanted pregnancies because these eliminate contraceptive failure caused by user error (Frost et al. 2007; Kost et al. 2008; Madden et al. 2011).

## Figures and Tables

**Figure A1:**
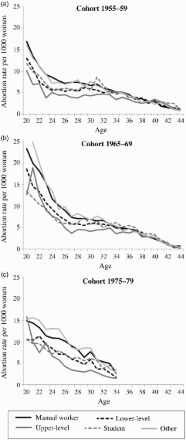
The number of first abortions for social reasons per 1,000 women of the same age and occupational group in Finland

*Source*: As for Figure 1.

## Appendix

As a robustness check, I conducted the discrete-time event-history analyses described in the paper by occupational group. The composition of the groups is as follows. Upper-level employees are women in managerial, professional, and related occupations. Lower-level employees have administrative and clerical occupations. Manual workers typically work in manufacturing or the distribution of goods and services. The ‘other’ category includes long-term unemployed, farmers, self-employed, pensioners, those outside the workforce, and those who do not belong to any of the other categories (Official Statistics of Finland 2013a).

The occupational composition of the population changed somewhat during the study period, although less dramatically than distribution by education. The proportion of upper-level employees at age 30 grew from 13 per cent in the 1950s cohort to 20 per cent in the latest cohort. Among women aged 20, students were the largest occupational group (around 40 per cent for the two earliest cohorts and 51 per cent for the latest) (Table A1).

Figure A1 shows that ‘other’ and manual worker groups had relatively more abortions than upper-level and lower-level employees across cohorts. For example, in the earliest cohort, manual workers had an average of 8 abortions per 1,000 at age 25 and upper-level employees 4 per 1,000. In the 1960s cohort the corresponding rates were 10 and 7, and in the 1970s cohort 11 and 6. The ‘other’ group had levels of abortion similar to those of the manual workers’ group. The differences by occupational group did not change substantially over time.

Upper-level and lower-level employees had lower odds of abortion than manual workers. For instance, lower-level employees had 10–12 per cent and upper-level employees 11–29 per cent lower odds of first abortion than manual workers at age 25–29, depending on cohort. The associations were stronger for younger women than for women in their 30s, but there was less variation across cohorts than for education (Table A2).

**Table A1:** Women's occupational status at ages 20, 25, and 30 by cohort in Finland, weighted percentage and unweighted *N*

	1955–59			1965–69			1975–79		
Category	20^1^	25^1^	30^1^	20^1^	25^1^	30^1^	20^1^	25^1^	30
Manual worker	22.6	24.8	21.2	19.1	19.7	17.4	15.1	20.4	15.8
Lower-level employee	25.3	41.8	44.6	24.8	36.2	34.6	13.5	31.9	39.5
Upper-level employee	0.8	6.6	13.2	1.6	8.0	14.4	1.3	9.5	20.4
Student	39.1	12.1	3.8	41.1	16.8	7.4	50.9	19.1	6.2
Other	10.1	11.9	15.6	12.4	18.2	25.1	18.2	18.0	17.3
Missing	2.1	2.8	1.6	1.1	1.1	1.1	0.9	1.1	0.8
Total = 100% (*N*)	(102,014)	(101,090)	(100,554)	(95,592)	(95,944)	(96,462)	(58,227)	(58,706)	(59,149)

^1^Measured at age 20, 25, or 30 or the nearest year possible (see text). For that reason, values of *N* for SES are different from education (sometimes measured in different years).

*Source*: As for Table 1.

**Table A2:** Discrete-time event-history models for first abortion by age group and cohort in Finland. Hazard-odds ratios (HOR) with 95 per cent confidence intervals

	20–24		25–29		30–34		35+	
Age	HOR	CI 95%	HOR	CI 95%	HOR	CI 95%	HOR	CI 95%
Cohort 1955–59								
*Occupational group*								
Manual worker (ref.)	1.00		1.00		1.00		1.00	
Lower-level employee	0.77	(0.72–0.83)	0.89	(0.82–0.96)	0.96	(0.88–1.05)	1.06	(0.97–1.15)
Upper-level employee	0.71	(0.52–0.96)	0.89	(0.75–1.05)	0.88	(0.76–1.02)	0.97	(0.85–1.11)
Student	0.71	(0.66–0.76)	0.92	(0.82–1.02)	1.13	(0.95–1.35)	1.08	(0.91–1.28)
Other	0.87	(0.80–0.95)	0.95	(0.86–1.05)	0.95	(0.85–1.06)	0.99	(0.89–1.10)
Cohort 1965–69								
*Occupational group*								
Manual worker (ref.)	1.00		1.00		1.00		1.00	
Lower-level employee	0.78	(0.73–0.83)	0.90	(0.83–0.97)	1.01	(0.91–1.12)	1.07	(0.97–1.19)
Upper-level employee	0.73	(0.61–0.88)	0.88	(0.76–1.02)	0.98	(0.84–1.14)	1.09	(0.94–1.26)
Student	0.62	(0.58–0.66)	0.83	(0.75–0.91)	0.97	(0.83–1.12)	1.20	(1.04–1.39)
Other	1.00	(0.93–1.08)	0.94	(0.86–1.02)	0.99	(0.90–1.10)	1.04	(0.93–1.15)
Cohort 1975–79								
*Occupational group*								
Manual worker (ref.)	1.00		1.00		1.00			
Lower-level employee	0.87	(0.78–0.96)	0.88	(0.80–0.96)	0.87	(0.77–0.99)		
Upper-level employee	0.77	(0.58–1.02)	0.71	(0.61–0.83)	0.79	(0.66–0.95)		
Student	0.75	(0.69–0.82)	0.84	(0.76–0.92)	0.89	(0.73–1.08)		
Other	0.95	(0.87–1.05)	0.95	(0.87–1.05)	0.91	(0.79–1.04)		

*Notes*: All models were estimated separately by cohort and age group, and include age, education, occupational group, indicator for being childless, months since last birth and its quadratic term, parity, relationship status, place of residence, and immigration status.

*Source*: As for Table 1.
